# Intraokulare Entzündungen bei Brolucizumab-Anwendung: Patientenmanagement – Diagnose – Therapie

**DOI:** 10.1007/s00347-021-01321-8

**Published:** 2021-02-08

**Authors:** F. G. Holz, C. Heinz, A. Wolf, H. Hoerauf, U. Pleyer

**Affiliations:** 1 Universitäts-Augenklinik Bonn, Ernst-Abbe-Str. 2, 53127 Bonn, Deutschland; 2 Augenzentrum am St. Franziskus-Hospital Münster, Münster, Deutschland; 3 Klinik für Augenheilkunde, Universität Duisburg-Essen, Essen, Deutschland; 4 Klinik für Augenheilkunde am Universitätsklinikum Ulm, Ulm, Deutschland; 5 Augenklinik, Universitätsmedizin Göttingen, Göttingen, Deutschland; 6 Klinik für Augenheilkunde, Charité – Universitätsmedizin Berlin, Campus Virchow-Klinikum, Berlin, Deutschland

**Keywords:** Anti-VEGF-Therapie, Brolucizumab, Entzündung, Nebenwirkung, Vaskulitis, Anti-VEGF treatment, Brolucizumab, Inflammation, Side effect, Vasculitis

## Abstract

Der VEGF(„vascular endothelial growth factor“)-Inhibitor Brolucizumab ist seit Oktober 2019 in den USA und seit Februar 2020 in Europa zur Behandlung der neovaskulären altersabhängigen Makuladegeneration (nAMD) zugelassen. Grundlage der Zulassung bildeten die randomisierten, doppel-blinden Phase-III-Studien HAWK und HARRIER mit insgesamt 1817 Patienten. Hierbei zeigte Brolucizumab 6 mg (je nach Krankheitsaktivität alle 12 oder alle 8 Wochen verabreicht) eine nichtunterlegene Wirksamkeit in Bezug auf den bestkorrigierten Visus gegenüber Aflibercept 2 mg (alle 8 Wochen verabreicht). Erste Rückmeldungen zum Einsatz von Brolucizumab nach der Marktzulassung in den USA haben einzelne, z. T. schwerwiegende Fälle behandlungsassoziierter intraokularer Entzündungen mit retinaler Vaskulitis und/oder retinaler, vaskulärer Okklusion beschrieben, die teilweise zu einem schweren Visusverlust führten. Die Daten der Zulassungsstudien wurden daraufhin durch ein Safety Review Committee (SRC) unabhängig retrospektiv analysiert. Ziel der vorliegenden Publikation ist es, Anwendern eine Orientierungshilfe aus Autorensicht bei der Therapie einer Brolucizumab-assoziierten intraokularen Entzündung zu bieten. Von zentraler Bedeutung ist hierbei auch eine erweiterte Aufklärung der Patienten über Symptome einer intraokularen Entzündung. Obwohl die Fallserien und die HAWK/HARRIER-Daten es nicht abschließend beantworten, bleiben eine zu späte Detektion, eine unterdosierte antientzündliche Therapie oder eine unbedachte Wiederbehandlung mit Brolucizumab dem Verdacht ausgesetzt, Komplikationen zu verstärken. Ein Stopp der Brolucizumab-Therapie sollte grundsätzlich erfolgen, sobald es nach Gabe des Medikaments zu intraokularen Entzündungen mit oder ohne retinalen Vaskulitiden und oder Gefäßverschluss kam. Abhängig vom Schwerpunkt der Entzündung werden dem Behandler an die Leitlinien und Stellungnahmen angelehnte Empfehlungen für Diagnostik und Therapie dargestellt. Diese Übersichtsarbeit ersetzt nicht die fachgesellschaftlichen Stellungnahmen.

## Abstract (en)

The anti-vascular endothelial growth factor (anti-VEGF) agent brolucizumab has been approved in the USA in October 2019 and in Europe in February 2020 for the treatment of neovascular age-related macular degeneration (nAMD). The approval was based on the randomized, double-blind phase III studies HAWK and HARRIER with a total of 1817 patients. Brolucizumab 6 mg (administered every 12 or 8 weeks depending on the activity of the disease) showed a non-inferior efficacy in terms of best-corrected visual acuity compared to aflibercept 2 mg (administered every 8 weeks). Initial reports on the use of brolucizumab after its approval in the USA indicated a safety signal of rare adverse events termed as retinal vasculitis and/or retinal vascular occlusion that may result in severe loss of vision. Typically, these events occurred in the presence of intraocular inflammation (IOI). A safety review committee (SRC) subsequently carried out an independent analysis of data from the pivotal studies. This article sets out the current state of knowledge and aims to provide users with orientation—from the authors’ perspective—in treating brolucizumab-associated IOI. It appears mandatory to provide patients with information about possible symptoms of IOI. Even though the case reports and the SRC review of HAWK/HARRIER may not yet provide sufficient evidence for any final conclusions, it seems crucial to educate patients about signs and symptoms to ensure an early detection and diagnosis in cases of IOI. Once a patient is diagnosed with IOI, retinal vasculitis, and/or retinal vascular occlusive events, physicians should act promptly with an adequate and intensive anti-inflammatory treatment and brolucizumab treatment should be discontinued. It is important to note that these recommendations are primarily based on the authors’ expert opinions and should be considered as guidance in managing these events rather than a formal protocol or guidelines.

## Handlungsempfehlungen bei der Verwendung von Brolucizumab zur Behandlung der neovaskulären AMD^a^

- Das für Brolucizumab bestehende Risiko einer retinalen Vaskulitis und/oder eines retinalen Gefäßverschlusses mit der möglichen Folge eines hochgradigen, irreversiblen Visusverlustes, muss klarer Bestandteil der Aufklärung der zu behandelnden Patienten sein [5, 13]. Es handelt sich um ein spezifisches Risiko bei Verwendung von Brolucizumab.
- Die Aufklärung sollte neben den bisherigen Risiken und Symptomen der infektiösen Endophthalmitis auch explizit Beschwerden wie Rötung, Glaskörpertrübung und Visusminderung auch im späteren Verlauf einschließen in Differenzierung zur bekannten typischen zeitlichen Manifestierung einer Post-IVOM-Endophthalmitis, die zumeist in den ersten 3 Tagen symptomatisch wird. Bisherigen Beobachtungen zufolge traten unerwartete Ereignisse im Spätverlauf (Durchschnitt 25 Tage [3 bis 63 Tage] post injectionem) auf [26]; 48 % der intraokularen Entzündungen ereigneten sich während der ersten 3 Monate und insgesamt 74 % während der ersten 6 Monate nach Therapiebeginn. Danach war das Auftreten entsprechend seltener [9, 26].
- Wie vor jeder Anti-VEGF-IVOM: Untersuchung des Patienten auf Zeichen einer aktiven oder abgelaufenen intraokularen Entzündung [13]. Zusätzlich anamnestische Hinweise auf zurückliegende (intra)okulare Entzündungen beachten [6–8].
- Liegen Hinweise auf eine intraokulare Entzündung nach IVOM mit Brolucizumab vor: Entzündungsschwerpunkt und -intensität (Vorderkammerzellen, Glaskörpertrübung, retinale Vaskulitis und/oder retinaler Gefäßverschluss) bestimmen (s. Anhang). Stets Untersuchung in Mydriasis. Bei intraokularer Entzündung mit Beteiligung des hinteren Augensegmentes: Bildgebung mit Fundusfotografie, optischer Kohärenztomographie (OCT), Fluoreszenzangiographie (FLA) einschließlich der Peripherie zur Detektion von Vaskulitiszeichen. Bei Patienten, bei denen diese Ereignisse (d. h. intraokulare Entzündungen, retinale Vaskulitis und/oder retinaler Gefäßverschluss) auftreten, sollte die Behandlung mit Brolucizumab abgebrochen und die Ereignisse sollten umgehend behandelt werden [13]. Eine rasche und ausreichend starke, antientzündliche Therapie erscheinen hierbei entscheidend [2, 3]. Ein Stopp der Brolucizumab-Therapie sollte deshalb grundsätzlich erfolgen, sobald es nach Gabe des Medikaments zu intraokularen Entzündungen mit oder ohne retinale Vaskulitiden und oder Gefäßverschluss kommt. Die Gabe von Brolucizumab ist im Falle des Auftretens von intraokularen Entzündungen oder bei Überempfindlichkeit gegen den Wirkstoff kontraindiziert [3, 13].
- Bei intraokularer Entzündung: antiinflammatorische Therapie gemäß Leitlinien der deutschen Fachgesellschaften „Uveitis anterior“, „intermedia“ und „posterior“ [6–8].

^a^Diese Übersichtsarbeit entspricht Expertenmeinung und ersetzt nicht die fachgesellschaftlichen Stellungnahmen.

In den USA wurde Brolucizumab im Oktober 2019 [10] zur Therapie der neovaskulären altersabhängigen Makuladegeneration (nAMD) zugelassen, im Februar 2020 folgte die Zulassung in Europa [13]. In den Phase-III-Zulassungsstudien HAWK und HARRIER zeigte der VEGF(„vascular endothelial growth factor“)-Inhibitor Brolucizumab (8- oder 12-wöchentliche Gabe) im Vergleich mit Aflibercept (8-wöchentliche Gabe^2^) nach 48 Wochen eine Nichtunterlegenheit in Bezug auf den Visus [12]. Bei Patienten ohne Krankheitsaktivität kann nach den ersten 3‑monatlichen Injektionen eine Behandlung alle 12 Wochen (3 Monate) in Betracht gezogen werden [13], da sich in den Zulassungsstudien in Bezug auf die Krankheitsaktivität ein etwas stärkerer flüssigkeitsreduzierender Effekt in der optischen Kohärenztomographie (OCT) von Brolucizumab gegenüber Aflibercept zeigte [12], wobei die längeren Injektionsintervalle durch das Studiendesign mitverursacht waren [12, 25].

Bezüglich unerwünschter Wirkungen lagen gegenüber bisher angewendeten Anti-VEGF-Wirkstoffen in der Zulassungsphase vergleichbare Risiken für intraokulare Entzündungen vor: 2–4 % [14, 18, 22]. Unerwartet waren dagegen Meldungen bei der American Society of Retina Specialists (ASRS) im Februar 2020 über einzelne, mit der Brolucizumab-Behandlung assoziierte nicht-/okklusive retinale Vaskulitiden, die in den üblichen MedDRA(Medical Dictionary for Regulatory Activities)-Klassifizierungen zur einheitlichen Kodierung von unerwünschten Ereignissen in klinischen Studien zuvor so nicht erfasst worden waren [4, 16, 21, 26]. Retinale Vaskulitiden oder Gefäßverschlüsse hingegen sind vereinzelt bereits in der Vergangenheit unter Anti-VEGF-Therapie berichtet worden [11, 15, 17, 18], jedoch nicht in der nun unter Brolucizumab beobachteten Häufung. Daraufhin beauftragte Novartis ein unabhängiges, externes Safety Review Committee (SRC) mit der Analyse und Bewertung [1]. Außerdem wurde das SRC damit betraut, retrospektiv die klinischen Daten und Befunde bildgebender Verfahren (Fundusaufnahmen, OCT-Dokumentation, Fluoreszenzangiographie [FLA]) aus den Zulassungsstudien einer erneuten gründlichen Prüfung zu unterziehen [9, 23]. Basierend auf dem Review der Post-Marketing-Fälle durch das SRC hat Novartis in Zusammenarbeit mit den Behörden (u. a. Food and Drug Administration [FDA] und European Medicines Agency [EMA]) die Aktualisierung der Fachinformationen angestoßen, die seit Juni (FDA) bzw. September (EMA) 2020 verfügbar sind und auf die Risiken von retinaler Vaskulitis und/oder retinalem Gefäßverschluss typischerweise in Verbindung mit einer intraokularen Entzündung hinweisen [1, 9, 10, 19, 23].

## SRC-Analyse der Daten aus den Zulassungsstudien

Aus den Studien HAWK und HARRIER war die Inzidenz der intraokularen Entzündung mit 4,4 % (Brolucizumab 6 mg) berichtet worden [4, 9, 23]. Das SRC hat alle potenziellen Fälle mit intraokularer Entzündung dieser Head-to-Head-Studien (einschließlich des Brolucizumab 3 mg^3^-Arms der HAWK-Studie) mit den Post-Marketing-Fällen aus den USA verglichen. Es wurden aus den Studien nicht nur die eindeutigen, sondern auch alle fraglichen Fälle mit intraokularer Entzündung ausgewertet. Während in einigen Fällen die entzündlichen Veränderungen nicht bestätigt wurden, kamen neue in der SRC-Bewertung hinzu, sodass mit 4,6 % eine vergleichbare Inzidenz wie in den 48-Wochen-Daten (4,4 %) gefunden wurde [4, 9, 23]. Wie das SRC jedoch feststellte, war die beobachtete Inzidenz sowohl der retinalen Vaskulitis (SRC-Bericht: 3,3 %) als auch des retinalen Gefäßverschlusses (SRC-Bericht: 2,1 %) höher als von den Studienärzten berichtet [9, 23].

Bezüglich funktioneller Ergebnisse konnte das SRC in den Zulassungsstudien keinen Unterschied für das Risiko eines Visusverlustes zwischen den Brolucizumab- und Aflibercept-behandelten Patienten feststellen [4, 9, 12, 23]: Die Inzidenz zumindest moderater Sehverluste (≥ 15 Buchstaben) durch eine mit Brolucizumab assoziierte intraokulare Entzündung liegt im Beobachtungszeitraum bei 0,7 % (8 von 1088 Patienten). Bei isolierter Betrachtung der Patienten mit intraokularer Entzündung erhöhte sich das Auftreten einer entsprechenden Visusminderung deutlich (8 von 50 Patienten, entsprechend 16 %). Als klinisch wichtiger Hinweis wurde durch das SRC auch die zeitliche Verteilung der Brolucizumab-assoziierten Uveitiden dokumentiert: 48 % traten in den ersten 3 Monaten und 74 % kumulativ in den ersten 6 Monaten nach Therapiebeginn auf. Von Monat 12 bis 18 traten nur noch 12 % der beobachteten Entzündungsreaktionen auf [9, 23].

## Ausprägungen der intraokularen Entzündung

In einer retrospektiven Untersuchung der nach Markteinführung in den USA gemeldeten Fälle lag der Ort der intraokularen Entzündung bei 8 (31 %) Augen anterior, bei 7 (27 %) Augen posterior und bei 9 (35 %) Augen sowohl anterior als auch posterior [26]. Besondere Aufmerksamkeit muss auf die retinale Vaskulitis gelenkt werden, die mit oder ohne Okklusion (v. a. der arteriellen Gefäße) auftreten kann, wie auch den hilfreichen, exemplarischen Abbildungen der veröffentlichten Fallberichte zu entnehmen ist [4, 26].

Bisherigen Beobachtungen zufolge waren bei dieser Form der Vaskulopathie zuvor typischerweise unspezifische Zeichen einer anterioren Uveitis (z. B. Hornhautendothelpräzipitate, intraokulare Zellen/Flare) vorangegangen, sodass zunächst eine engmaschige Kontrolle aller Patienten mit jeglichen Zeichen einer intraokularen Entzündung angeraten wird [2–4, 6]. Die meisten Patienten mit intraokularer Entzündung entwickelten während des Beobachtungszeitraums keine visusbedrohende Form einer Vaskulitis [26].

Verläufe als anteriore oder intermediäre Uveitis wiesen keinen Einfluss auf die Funktion (Sehschärfe) – im Sinne einer Verschlechterung gegenüber der Sehfunktion vor Entzündung – auf. Unter den Patienten, die eine Vaskulitis aufwiesen, wurde der Visus ebenfalls nicht immer beeinträchtigt. Funktionelle Konsequenzen traten hingegen v. a. dann ein, wenn retinale Gefäßäste zur Versorgung der Makula betroffen waren. Dies führte in vereinzelten Fällen zu einer schweren Visusminderung (≥30 Buchstaben auf der ETDRS[Early Treatment Diabetic Retinopathy Study]-Sehtafel) [4, 9, 23].

Der den intraokularen Entzündungen zugrunde liegende Mechanismus ist derzeit noch nicht bekannt. Eine unmittelbar toxische Genese oder eine Reaktion auf die Injektion selbst sind aufgrund des verzögerten Auftretens eher unwahrscheinlich. Die Verläufe der Patienten mit einer okklusiven Vaskulitis erinnern jedoch an Bilder, wie sie auch bei Patienten mit Morbus Behçet gesehen werden. Dies könnte dafür sprechen, dass autoimmunologische oder autoinflammatorische Mechanismen beteiligt sind.

## Patientenaufklärung

Hinsichtlich der Früherkennung einer Brolucizumab-assoziierten intraokularen Entzündung ist die dezidierte Patientenaufklärung inklusive der in der Fachinformation [13] und im Risikomanagementplan(RMP)-Material [5] spezifizierten Risiken und Hinweise von vorrangiger Bedeutung [2, 3]. Neben der standardmäßig durchgeführten Aufklärung über die Symptome einer bakteriellen Endophthalmitis ist im Vorfeld der Brolucizumab-Therapie die Aufklärung über die nichtinfektiösen Entzündungsprozesse und die für Brolucizumab spezifischen retinalen Vaskulitiden und Gefäßverschlüsse geboten [13]. Dies betont die Leitsymptome der unterschiedlichen Entzündungsformen. Zeichen der Uveitis anterior können Schmerzen, Rötung und Sehverschlechterung sein [6]. Typische Zeichen einer Uveitis intermedia sind Glaskörpertrübung, Sehverschlechterung und ggf. Metamorphopsien [7]. Grundsätzlich gilt es, Patienten dafür zu sensibilisieren, sich schon bei leichten Veränderungen und Symptomen unmittelbar beim Augenarzt vorzustellen, da die hier beobachteten Veränderungen deutlich subtiler sein können und sich von jenen einer klassischen bakteriellen Endophthalmitis unterscheiden. Allerdings können sich die initialen Symptome ähneln und die Differenzierung erschweren, bzw. vice versa kann eine okklusive Vaskulitis auch mit einer ausgeprägten Entzündungsreaktion einhergehen, die morphologisch von einer (beginnenden) Endophthalmitis nicht immer sicher unterscheidbar ist.

Patienten sind außerdem darauf hinzuweisen, dass die erhöhte Aufmerksamkeit nicht nur für die erste Woche nach der intravitrealen operativen Medikamentengabe (IVOM) gilt, sondern den gesamten Zeitraum bis zur nächsten Injektion betrifft. Denn während Symptome einer bakteriellen Endophthalmitis in der Regel innerhalb der ersten Tage nach der IVOM auftreten, können sich die mit Brolucizumab assoziierten nichtinfektiösen intraokularen Entzündungen über einen prolongierten Zeitraum entwickeln [4, 26].

## Diagnostik zur IVOM-Indikationsstellung

Die Diagnostik zur Indikationsstellung einer IVOM soll die augenärztliche Untersuchung mit bestkorrigiertem Visus, Funduskopie in Mydriasis, OCT und – zumindest bei der Erstindikationsstellung – FLA umfassen [25].

Eine bestehende aktive intraokulare Entzündung bzw. Kontraindikationen sollten im Vorfeld einer Anti-VEGF-Injektion zur Behandlung einer nAMD ausgeschlossen werden. Um Patienten mit Zeichen einer abgelaufenen intraokularen Entzündung bei Therapiebeginn bzw. vor einer wiederholten IVOM von der Behandlung mit Brolucizumab generell auszuschließen, gibt es aktuell zu wenig Evidenz. Sie sollte aber nur nach sorgfältiger Nutzen-Risiko-Abwägung erfolgen, da die Abgrenzung zwischen Brolucizumab-assoziierter Entzündung und Reaktivierung der vorbestehenden Uveitis schwierig ist. Sind nach einer Brolucizumab-IVOM auftretende entzündliche Reaktionen mutmaßlich medikamenteninduziert, muss die Behandlung mit Brolucizumab abgebrochen werden und bei Behandlungsbedürftigkeit der nAMD ggf. auf ein alternatives Therapiekonzept bzw. Anti-VEGF-Präparat gewechselt werden, da die Gabe von Brolucizumab im Falle einer intraokularen Entzündung oder Überempfindlichkeit gegen den Wirkstoff kontraindiziert ist [3, 13].

## Zeitpunkt der IVOM-Nachsorge

Gemäß Stellungnahme der deutschen Fachgesellschaften sollte innerhalb der ersten Woche nach einer IVOM eine Kontrolluntersuchung zur Beurteilung entzündlicher Reaktionen erfolgen [25]. Die EBM-Ziffer „postoperative Nachbehandlung“ schließt die binokulare Untersuchung des Augenhintergrundes in Mydriasis (06333) mit ein. Eine bakterielle Endophthalmitis tritt – im Gegensatz zu einer nichtinfektiösen intraokularen Entzündung – typischerweise um den 3. bis 7. Tag nach IVOM auf. Treten also innerhalb der ersten 7 Tage nach einer Brolucizumab-IVOM entsprechende Symptome oder Entzündungszeichen auf, könnte dies eher auf eine bakterielle Endophthalmitis anstelle einer nichtinfektiösen intraokularen Entzündung hinweisen. Sollte keine klare Differenzialdiagnose gestellt werden können, sollte im Zweifelsfall in Betracht gezogen werden, das Ereignis sowohl antibiotisch (und/oder chirurgisch) als auch zugleich antiinflammatorisch zu behandeln.

Der prolongierte Zeitraum, in dem sich die mit Brolucizumab assoziierten intraokularen Entzündungen entwickeln können, gibt keinen Anlass dazu, vom bislang üblichen Vorgehen bei der IVOM-Nachsorge abzuweichen. Dies betrifft insbesondere zusätzliche Nachuntersuchungen bei asymptomatischen Patienten. Er hebt aber nochmals den hohen Stellenwert der Patientenaufklärung hervor – insbesondere mit dem Hinweis, bei Auftreten entsprechender (auch geringfügiger) Symptome und Zeichen sofort einen Augenarzt zu konsultieren –, auch wenn die IVOM bereits längere Zeit zurückliegt. Auch die nachbetreuenden Augenärzte sollten bezüglich dieser Vorsorgemaßnahme sensibilisiert werden.

## Diagnose der Uveitis und Vaskulitis

**Figure 1:**
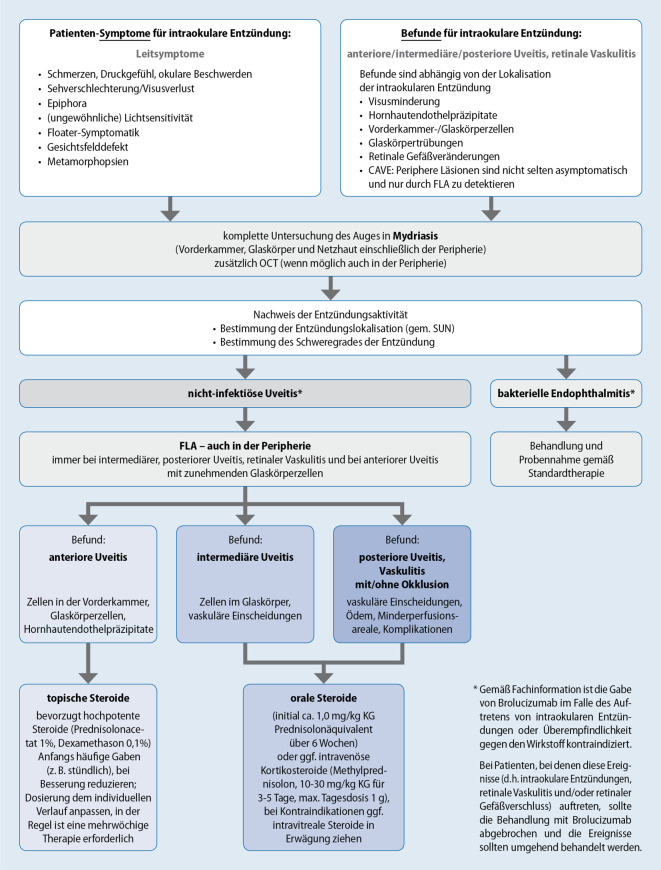

Zum Umgang mit unerwünschten entzündlichen Ereignissen bei Patienten mit nAMD unter Brolucizumab-Behandlung kann das in Abb. 1 dargestellte Flussdiagramm herangezogen werden.

Für die Diagnose und Bestimmung des Schweregrads intraokularer Entzündungen wird die international akzeptierte (anatomische) Uveitis-Klassifikation zugrunde gelegt (Tab. 1; [6, 20]).

**Table 1:** 

Typ	Primärer Entzündungsort^a^	Schließt mit ein
Anteriore Uveitis	Vorderkammer	Iritis Iridozyklitis Anteriore Zyklitis
Intermediäre Uveitis	Glaskörper	Pars planitis Posteriore Zyklitis Hyalitis
Posteriore Uveitis	Netzhaut oder Aderhaut	Fokale, multifokale oder diffuse Choroiditis, Retinochoroiditis, Retinitis, Neuroretinitis
Panuveitis	Vorderkammer, Glaskörper und Netzhaut oder Aderhaut	–

^a^Klinisch ermittelt und angelehnt an die anatomische Klassifikation der International Uveitis Study Group

Zur Diagnose und Therapie der Brolucizumab-assoziierten intraokularen Entzündung können die Leitlinien „Uveitis anterior“, „Uveitis intermedia“ und „Uveitis posterior“ der deutschen Fachgesellschaften herangezogen werden [6–8].

Dabei können die folgenden 3 Szenarien unterschieden werden:

1. Anteriore Uveitis: Entzündung auf die Vorderkammer begrenzt
2. Intermediäre Uveitis:
  Entzündungsschwerpunkt im Glaskörper
  Gefäßbeteiligung möglich
  Netzhaut nicht betroffen
3. Posteriore Uveitis/Panuveitis mit Vaskulitis und/oder Gefäßokklusion, Netzhaut- und Aderhautbeteiligung möglich.

## Ziele des diagnostischen und therapeutischen Vorgehens

Ziele des diagnostischen und therapeutischen Vorgehens bei Brolucizumab-assoziierter intraokularer Entzündung sind [6]:

- Nachweis der Entzündungsaktivität
- Bestimmung der Lokalisation und der Schwere der Entzündung (gemäß SUN [„standardization of uveitis nomenclature“])
- Erhalt oder Wiederherstellung der Sehfähigkeit
- Vermeidung und möglichst frühzeitige Behandlung von Komplikationen

Zur weiteren Differenzialdiagnose sei auf die Leitlinien zur Uveitis verwiesen [6–8].

## Symptome

Typische Symptome der nichtinfektiösen Uveitiden sind Visusminderung (Infiltration mit Entzündungszellen, myoper oder hyperoper Shift), Photopsien, Skotome und Glaskörpertrübungen [6–8].

Die Symptomatik der intermediären/posterioren Uveitis ist abhängig von der Lokalisation der Entzündung, wobei periphere Läsionen nicht selten asymptomatisch verlaufen und Symptome und Befunde einer akuten Vorderkammerentzündung (Rötung, Schmerzen) fehlen.

## Befunde und Handlungsempfehlungen

### Anteriore Uveitis

Liegen bei der Spaltlampenuntersuchung Hornhautendothelpräzipitate und/oder Zellen in der Vorderkammer vor, ist die Untersuchung des Auges in Mydriasis zwingend notwendig (Glaskörper- und Netzhautbeurteilung einschließlich der Peripherie), um eine Vaskulitis sicher auszuschließen. Die semiquantitative Graduierung des Reizzustandes (Vorderkammerzellen, Tyndall) zur Verlaufskontrolle wird empfohlen (Tab. 2) [6]. Die Durchführung einer FLA ist bei anteriorer Uveitis nicht erforderlich. Darüber hinaus können weitere Entzündungsmerkmale im Einzelfall differenzialdiagnostische Hinweise bieten [24]. Im Rahmen eines „spill over“ können bei anteriorer Uveitis einzelne Glaskörperzellen im vorderen Glaskörperanteil vorliegen; diese sollten bei der Verlaufsbeobachtung nicht zunehmen, andernfalls jedoch eine weitergehende Untersuchung (FLA, s. unten) nach sich ziehen.

### Intermediäre Uveitis

Primärer Ort der Entzündung bei einer Uveitis intermedia sind der Glaskörper und/oder die Pars plana (± periphere Vv. Gefäßeinscheidungen ± ggf. Makulaödem) (Tab. 3) [7]. Im Rahmen der Diagnose werden folgende Untersuchungen empfohlen [7]:

- Sehschärfenbestimmung, Tonometrie
- Spaltlampenuntersuchung der vorderen und mittleren Augenabschnitte zur Beurteilung des Schweregrades der Entzündung in Vorderkammer und Glaskörper
- binokulare Untersuchung des Glaskörpers und des gesamten Augenhintergrundes in Mydriasis mit semiquantitativer Gradeinteilung der Glaskörpertrübung bei der binokularen indirekten Fundoskopie

Bei Verdacht auf eine medikamentenassoziierte intraokulare Entzündung ist die Indikation für eine FLA gegeben. Aufnahmen auch der Netzhautperipherie sind essenziell. Die Verlaufsbeurteilung der intermediären Uveitis erfolgt ebenfalls obligat in Mydriasis.

### Uveitis posterior

Zur Beurteilung von Netzhaut- und Aderhautbeteiligung sowie struktureller Komplikationen liefert die FLA wichtige Informationen, die auch im Verlauf relevant sein können [8]:

- Nachweis von entzündlichen Veränderungen in Netzhaut, Aderhaut, Papille und Netzhautgefäßen
- Nachweis typischer Komplikationen, wie z. B. Makulaödem, Papillenödem, Gefäßverschluss
- Beurteilung der Entzündungsaktivität (z. B. entzündliche Läsionen), z. B. Gefäßleckage, RPE(retinales Pigmentepithel)-Alterationen, Makulaödem
- Diagnose eines Makulaödems, das ggf. mittels OCT nicht nachweisbar sein kann

### Retinale Vaskulitis

Der retinalen Vaskulitis muss aufgrund ihres potenziell visusbedrohenden Verlaufs hinsichtlich frühzeitiger Diagnose und Intervention besondere Beachtung geschenkt werden. Es handelt sich um eine Sonderform der intraokularen Entzündung, die nicht mit einer klassischen Uveitis gleichzusetzen ist und in der SUN-Klassifikation nicht eingeschlossen ist. Die seltenen retinalen Vaskulitiden unter Brolucizumab (Inzidenzrate nach Markteinführung für retinale Vaskulitis und/oder retinale Gefäßverschlüsse [Stand 20.11.2020]: 15,47 pro 10.000 Injektionen; regelmäßig aktualisierte Inzidenz einsehbar unter [9]) präsentierten sich bisher typischerweise als periphere, segmentale Vaskulitiden, die durch segmentale Gefäßeinscheidungen gekennzeichnet sind. Das Spektrum reicht von der peripheren Gefäßbeteiligung bis hin zu arteriellen Verschlüssen. Betreffen diese die Versorgung von Sehnerv oder Makula, sind schwere funktionelle Störungen mit Visusminderung und Gesichtsfelddefekten möglich. Zu den weiteren möglichen Zeichen einer Brolucizumab-assoziierten ischämischen Vaskulitis zählen Cotton-Wool-Spots und intraretinale Hämorrhagien [2–4].

## Therapie

Angaben über die Dauer der Therapie einer Brolucizumab-assoziierten intraokularen Entzündung lassen sich zum jetzigen Zeitpunkt nicht verlässlich machen. Entscheidend ist das Ansprechen auf die unten aufgeführten Therapiestrategien.

### Uveitis anterior

Eine auf die Vorderkammer begrenzte Entzündung sollte immer behandelt werden und lässt sich mit topischer Therapie in der Regel gut beherrschen. Die Leitlinie „Uveitis anterior“ gibt zur Therapie des akuten Schubes die folgende Empfehlung [6]:

- Kortikosteroide; bevorzugt hochpotente Wirkstoffe (Prednisolonacetat 1 %, Dexamethason 0,1 %)
- anfangs häufige Gaben (z. B. stündlich), bei Besserung reduzieren; Dosierung dem individuellen Verlauf anpassen; in der Regel ist eine mehrwöchige Therapie erforderlich
- in Abhängigkeit von der Ausprägung der Inflammation kann eine medikamentöse Mydriasis erforderlich sein
- bei Steroidrespondern oder auch in der Ausschleichphase können ggf. schwächere Wirkstoffe verwendet werden
- Kontrolle des Augeninnendruckes zum Ausschluss einer steroid- oder entzündungsinduzierten okulären Hypertension; ggf. drucksenkende Medikation (z. B. Betablocker, Karboanhydrasehemmer)

Bei fehlendem Ansprechen auf eine topische Therapie oder bei Ausdehnung des Inflammationsgeschehens auf den Glaskörperraum und Beteiligung des hinteren Augenabschnitts (v. a. bei FLA-gesicherter Vaskulopathie) ist die systemische Behandlung indiziert.

### Uveitis intermedia, Uveitis posterior, retinale Vaskulitis

Die Behandlung einer Uveitis intermedia erfolgt immer bei Vorliegen eines Makulaödems oder einer ausgeprägten Vitritis. Findet sich eine intermediäre Uveitis mit Vaskulitis, eine posteriore Uveitis oder eine solitäre retinale Vaskulitis nach Brolucizumab-Injektion, ist primär eine systemische Therapie mit Kortikosteroiden indiziert [7].

Die in der Leitlinie „Uveitis intermedia“ aufgeführten Empfehlungen sollten zur Therapie einer posterioren Uveitis oder einer retinalen Vaskulitis angewendet werden [7]:

- Im akuten Stadium der intermediären Uveitis: orale (initial ca. 1 mg/kgKG Prednisolonäquivalent) oder ggf. intravenöse Kortikosteroide (Methylprednisolon, 10–30 mg/kgKG für 3 bis 5 Tage, maximale Tagesdosis 1 g) über ca. 6 bis 12 Wochen; schrittweise reduzieren bis ggf. zu einer Erhaltungsdosis von ca. 0,1 mg/kgKG
- Höhere Dosierungen und eine länger dauernde Dosisreduktion können im Einzelfall gerechtfertigt sein
- Bisher liegen keine Erfahrungen zu immunmodulierenden Wirkstoffen („disease-modifying anti-rheumatic drugs“ [DMARDs]) vor, die ggf. im Einzelfall verwendet werden können

Bei Kontraindikation einer systemischen Kortikosteroidtherapie kann eine IVOM mit einem Steroid („off-label“) erwogen werden.

Bei sekundärer retinaler Neovaskularisation: sektorielle Photokoagulation im Ischämieareal; Vitrektomie wird nicht empfohlen, sondern nur bei intravitrealer Hämorrhagie. Bei starker Glaskörperbeteiligung kann eine Pars-plana-Vitrektomie erwogen werden.

## Fazit

- Die Aufklärung der nAMD-Patienten über Symptome einer intraokularen Entzündung zählt aufgrund des Risikos einer bakteriellen Endophthalmitis nach Anti-VEGF-IVOM bereits zur regelhaften Routine. Die Möglichkeit einer Brolucizumab-induzierten Entzündung bis hin zur retinalen Vaskulitis und/oder einem retinalen Gefäßverschluss erfordert eine umfangreiche Information und Aufklärung der Patienten, die über Symptome und Risiken der Endophthalmitis nach IVOM hinausgeht.
- Die Endophthalmitis manifestiert sich in der Regel zeitnah nach der Injektion. Demgegenüber kann die Brolucizumab-assoziierte nichtinfektiöse intraokulare Entzündung zu jedem Zeitpunkt auftreten und wurde bisher überwiegend im Zeitverlauf (Durchschnitt 25 Tage; Mehrzahl innerhalb von 6 Monaten nach initialer Injektion) beobachtet.
- Patienten sind deshalb explizit darauf hinzuweisen, auch bei geringfügigen Symptomen und Zeichen unabhängig vom Zeitpunkt ihres Auftretens nach einer Injektion unverzüglich ihren Augenarzt aufzusuchen [2, 3, 5, 13, 26].
- Bei Patienten, bei denen diese Ereignisse (intraokulare Entzündung, retinale Vaskulitis, retinaler Gefäßverschluss) auftreten, sollte die Behandlung mit Brolucizumab abgebrochen und die Ereignisse sollten umgehend behandelt werden [2, 3, 13]. Eine rasche und ausreichend starke antientzündliche Therapie erscheint hierbei entscheidend [2, 3]. Die Gabe von Brolucizumab ist im Falle des Auftretens von intraokularen Entzündungen kontraindiziert [3, 13].
- Zur Diagnose und Therapie der intraokularen Entzündungen können die Uveitis-Leitlinien der Fachgesellschaften herangezogen werden [6–8].

## Anhang

### Klassifikations- und Aktivitätskriterien bei intraokularer Entzündung

#### Graduierung des Reizzustandes in der Vorderkammer

Zur Bestimmung der zellulären Infiltration in der Vorderkammer hat sich die klinische Einschätzung bewährt. Im 2 mm hohen und 1 mm breiten Spaltlampenstrahl werden die Zellen über 15 s gezählt.

#### Graduierung des Reizzustandes im Kammerwasser

**Table 2:**

Grad	Vorderkammerzellen pro Feld	Trübung (Tyndall-Effekt)
0	< 1	Keine Trübung
0,5+	1–5	Leichte Trübung
1+	6–15	Leichte Trübung
2+	16–25	Moderate Trübung (Iris und Linsendetails klar)
3+	26–50	Ausgeprägte Trübung (Iris und Linsendetails verschwommen)
4+	> 50	Massive Trübung (Fibrin oder plastisches Kammerwasser)

Das Kammerwasser ist unter physiologischen Bedingungen klar und zellfrei. Bei Veränderungen der Blut-Kammerwasser-Schranke treten Proteine in das Kammerwasser ein. Eine semiquantitative Beurteilung bei Biomikroskopie und schräger Spalteinstellung erfolgt nach Hogan.

#### Graduierung des Reizzustandes im Glaskörper

Zur Beurteilung des Glaskörperbefundes wurde ebenfalls eine quantitative Einschätzung vorgeschlagen (s. Graduierung im Kammerwasser). Klinisch hat sich eher die Einschätzung nach dem Funduseinblick bei indirekter Ophthalmoskopie („NEI“-Skala) mit 6 Graden der Glaskörpertrübung bewährt.

**Table 3:**

Beschreibung	Grad
Klar	0
Geringe Trübung	0,5
Papille und Gefäße klar, Nervenfaserschicht verschwommen	1
Papille und Gefäße verschwommen	2
Papille sichtbar	3
Papille nicht sichtbar	4

### Retinale Vaskulitis: Klassifikations‑/Aktivitätskriterien

Bisher existieren keine allgemein akzeptierten Klassifikations- und Aktivitätskriterien für die retinale Vaskulitis [24].

#### Klassifikation

Klinisch hat sich eine morphologische Klassifikation bewährt (typ. klinische Krankheitsbilder):

##### Arterien:

Akute Netzhautnekrose (ARN), progressive äußere Netzhautnekrose (PORN), Syphilis, systemischer Lupus erythematodes (SLE), Aneurysma und Neuroretinitis (IRVAN) und Churg-Strauss-Syndrom

##### Venen:

Intermediäre Uveitis, Sarkoidose, multiple Sklerose, Birdshot-Chorioretinitis, Tuberkulose, Morbus Eales

##### Sowohl Arterien als auch Venen:

Morbus Behçet, Granulomatose mit Polyangiitis (ehemals Morbus Wegener) „Frosted branch Angiitis“, rezidivierende Polychondritis, Morbus Crohn

#### Aktivitätskriterien

Um den Verlauf intraokularer Entzündung bei intermediärer und posteriorer Uveitis zu beurteilen, ist die Fluoreszenzangiographie (FLA) oft klinisch notwendig. Als Beurteilungskriterien der FLA werden herangezogen: Papillenhyperfluoreszenz, Makulaödem, Retina-Gefäßfüllung und/oder -Leckage, Kapillarleckage der Retinakapillaren, Retina-Nonperfusionareale, Okklusion, Neovaskularisation im Papillenbereich, Neovaskularisation anderer Regionen, punktförmige Leckagen und Retina „staining“ und/oder subretinales „pooling“.

## Footnotes

2 Aktuelle Zulassung von Aflibercept lässt Erhöhung des 8‑Wochen-Intervalls in 2‑ bis 4‑wöchigen Schritten zu.

3 Die 3‑mg-Dosierung von Brolucizumab ist in Europa nicht zugelassen.
